# Tetramethylpyrazine Suppresses Transient Oxygen-Glucose Deprivation-Induced Connexin32 Expression and Cell Apoptosis via the ERK1/2 and p38 MAPK Pathway in Cultured Hippocampal Neurons

**DOI:** 10.1371/journal.pone.0105944

**Published:** 2014-09-19

**Authors:** Gu Gong, Libang Yuan, Lin Cai, Maorong Ran, Yulan Zhang, Huaqu Gong, Xuemei Dai, Wei Wu, Hailong Dong

**Affiliations:** 1 Department of Anesthesia, General Hospital of Chengdu Military Area Command, Chengdu, Sichuan, China; 2 Department of Anesthesia, the Fourth Military Medical University Xijing Hospital, Xi’an, Shaanxi, China; University of Pecs Medical School, Hungary

## Abstract

Tetramethylpyrazine (TMP) has been widely used in China as a drug for the treatment of various diseases. Recent studies have suggested that TMP has a protective effect on ischemic neuronal damage. However, the exact mechanism is still unclear. This study aims to investigate the mechanism of TMP mediated ischemic hippocampal neurons injury induced by oxygen-glucose deprivation (OGD). The effect of TMP on hippocampal neurons viability was detected by MTT assay, LDH release assay and apoptosis rate was measured by flow cytometry. TMP significantly suppressed neuron apoptosis in a concentration-dependent manner. TMP could significantly reduce the elevated levels of connexin32 (Cx32) induced by OGD. Knockdown of Cx32 by siRNA attenuated OGD injury. Moreover, our study showed that viability was increased in siRNA-Cx32-treated-neurons, and neuron apoptosis was suppressed by activating Bcl-2 expression and inhibiting Bax expression. Over expression of Cx32 could decrease neurons viability and increase LDH release. Furthermore, OGD increased phosphorylation of ERK1/2 and p38, whose inhibitors relieved the neuron injury and Cx32 up-regulation. Taken together, TMP can reverse the OGD-induced Cx32 expression and cell apoptosis via the ERK1/2 and p38 MAPK pathways.

## Introduction

Ischemic brain injury is a principal pathology in survivors of ischemic stroke and cardiac arrest, two of the most significant diseases in the world. It can induce severe cognitive and motor dysfunction, neurodegenerative diseases and even sudden death [1]. Ischemic stress causes serious brain injury via various pathologic mechanisms including suppressed protein synthesis, neuronal apoptosis, and the release of neurotoxic substances [2]. Cerebral ischemia can lead to short- and long-term behavioral deficits that are associated with a reduction in the number of hippocampal pyramidal neurons [3]. Hence, many neuroprotective treatments for ischemic brain injury rely on these pathologic mechanisms. Traditional Chinese herbal medicine has been described in medicine systems as a neuroprotective treatment associated with ischemic brain injury.

Tetramethylpyrazine (TMP), a biologically active alkaloid extracted from Ligusticum chuanxiong Hort, has been widely used in China as a drug together with other Chinese herbal medicines for the treatment of various diseases. A great deal of pharmacological research has been done on this agent, mainly focused on its cardiovascular and cerebrovascular effects, anti-oxidation, neuroprotection, anti-fibrosis, anti-nociception, anti-inflammatory, and anti-neoplastic activity [4]. A previous study showed that TMP treatment promotes the expression of brain derived neurotrophic factor (BDNF) and basic fibroblast growth factor (bFGF) after severe brain injury in rats [5]. With ischemic stroke, TMP exhibited neuroprotective and anti-inflammatory effects in rats subjected to permanent cerebral ischemia [6]. Morphological studies have indicated that TMP has a protective effect on ischemic neuronal damage in hippocampus by regulating free radicals and free calcium [7]. However, scientific evidence related to its effectiveness or the precise mode of TMP’s neuroprotective action remains largely unclear.

Connexins (Cx) are a family of structurally related transmembrane proteins that assemble to form vertebrate gap junctions. There are 21 known connexins in the human genome, 11 of which are expressed in the central nervous system [8]. Connexin32 (Cx32) is expressed abundantly in mammalian brain but with differing cellular specificities. Cx32 and Cx36 gap junctions may contribute to the survival and resistance of GABAergic interneurons, thereby defining cell-specific patterns of global ischemia-induced neuronal death [9]. These observations raise the possibility that Cx32 gap junctions might play a role in the survival of hippocampal interneurons and the death of pyramidal neurons after ischemia. In the present study, we examined the effects of TMP on neuron injury induced by OGD in cultured hippocampal neurons and its potential mechanism.

## Materials and Methods

### Reagents

TMP (>99%) was obtained from Zelang Pharmaceutical Co. (Jiangsu, China). The Fluorescein isothiocyanate (FITC)-Annexin V/Propidium iodide (PI) apoptosis assay kit was from Bio-Rad (Hercules, CA). The enhanced chemiluminescence Western blot detection reagents were from Pierce (Rockford, USA). Dimethyl sulfoxide (DMSO), propidium iodide (PI) and 3-(4, 5-Dimethylthiazol-2-yl)-2, 5-diphenyltetrazolium bromide (MTT) were from Abcam (St. Cambridge, UK).

### Primary hippocampal neuron cultures

Animal experiments conformed to the guidelines issued by the Institute of General Hospital of Chengdu Military Area Command for Laboratory Animals. The present study was performed with approval from by the Animal Ethics Committee of the Institute of General Hospital of Chengdu Military Area Command. All surgery was performed under sodium pentobarbital anesthesia (Sigma, St. Louis, MO), and all efforts were made to minimize suffering. Primary culture of hippocampal neurons was isolated and cultured as previously described [10]. Briefly, primary hippocampal neurons were prepared from from embryonic day 18 (E18) Wistar rat brains. Neurons were plated on poly-D-lysine and laminin coated 6-well dishes at densities of 1×10^6^/well as previously described [11]. Neurons were grown at 37°C under a humidified atmosphere of 5% CO_2_ and 95% air in Neurobasal medium supplemented with B-27, glutamine (0.5 mM), glutamate (25 µM), and 1% penicillin/streptomycin, and then half-replaced twice every week. Culture cells were used after 14 days in vitro.

### Oxygen-glucose deprivation (OGD) model

OGD was induced as previously described [12], [13] with slight modification. The cells were fed with glucose-free DMEM and infused with 95% N_2_ and 5% CO_2_ at 37°C for 1 h. The maintenance medium was used to terminate OGD. Primary hippocampal neurons were maintained in DMEM containing 5% CO_2_ and 95% air. At the end of 4 h OGD, the medium was replaced, and cells were recovered in normal conditions for the next 21 h. In all experiments, the pH of the medium was maintained at 7.2 under OGD conditions.

### Treatments of TMP and inhibitors

TMP was solubilized in dimethyl sulfoxide (DMSO) and stored at −80°C. It was made up fresh each time and diluted in PBS to the desired concentration (10 and 20 µM). The concentration of DMSO in culture medium was used under 0.1% (v/v) without any effect on cell on its own. All drugs were applied from OGD to the end of experiments. Controls and OGD group received the same amount of DMSO. The following inhibitors were used: zVAD-fmk (caspase inhibitor 50 µM), U0126 (ERK1/2 inhibitor 5 µM), MAPK inhibitors SB203580 (p38 inhibitor 5 µM), and SP600125 (JNK inhibitor 5 µM) (Sigma-Aldrich). The inhibitors were added from 30 min before OGD to the end of experiments, and then control cells underwent the same procedures except for OGD.

### Cell viability and LDH release assay

Neuron viability was determined by measuring reduction of 3-(4,5-dimethylthia-zol-2-yl)-2,5-diphenyltetrazolium bromide (MTT). After 24 h of incubation, 10 µL of an MTT solution (5 mg/mL in phosphate buffered solution) were added to the wells and the plate incubated for a further 4 h. After incubation, the culture medium in each well was replaced with 150 µL of DMSO and the plates were shaken to dissolve the dark blue crystals (formazan). Absorbance was measured at 570 nm using an enzyme linked immunosorbent assay plate reader (Olympus, Tokyo, Japan), compared with the untreated neurons, and the percentage of viable neurons calculated. Each concentration was analyzed in triplicate and the experiment was repeated three times [14]. Cell membrane damage leading to cellular death was measured by elevation of lactate dehydrogenase (LDH) in the medium sample using a detection kit (Jiancheng Bioengineering Institute, Nanjing, China). LDH release into the cultured medium was determined using a colorimetric reaction reading of absorbance at 490 nm according to manufacturer’s protocol. The samples were measured in three replicates, and each experiment was repeated three times.

### Analysis of cell apoptosis

The apoptosis rate was measured using an annexin V-FITC/PI apoptosis detection kit and flow cytometry (Gibco, Rockville, MD). After 24 h of incubation, the neurons were washed twice with PBS and subjected to Annexin V-FITC and propidium iodide (PI) double staining as described in the manufacturer’s instructions. After 30 minutes at 37°C, the stained neurons were analyzed by flow cytometry and the rate of cell apoptosis was determined [15], [16].

### RT-PCR

At the end of the experiment, total RNA was isolated from the cells using the miRNeasy kit (Qiagen) according to the manufacturer’s protocol. Reverse transcription was achieved with the High Capacity cDNA Kit according to the manufacturer’s instructions. Quantitative PCR (qPCR) reactions were performed in a 7900HT Real-Time PCR System (Applied Biosystems, Foster City, CA) using SYBR Green Supermix (Invitrogen, Carlsbad, CA). Relative expression levels of genes were calculated using the 2^−ΔΔCT^ method and the house keeping gene β-actin was utilized as a control [17], [18]. The primer sequences used for qRT-PCR were as follows: Cx32 forward, 5’-CCCTGCAACTCATCTTGGTT-3’, Cx32 reverse 5’-ATTGCCCACACCCTCAATAA-3’[19].

### Western blot

At the end of the experiment, the total proteins were separately extracted from the hippocampal neuronal tissues or cultures according to previous methods. The protein concentration was measured using a BCA protein assay kit (Beyotime Institute of Biotechnology, Haimen, China) The same amount of protein from each sample was separated by sodium SDS-PAGE on a 12% gel and transferred to a nitrocellulose membrane. The following antibodies for Cx32, Bcl-2, Bax, ERK1/2, p-ERK1/2, p38, p-p38, JNK, p-JNK and β-actin (Abcam Cambridge, MA) were used and, subsequently, horseradish peroxidase-conjugated secondary antibodies (Invitrogen, Carlsbad, CA). Finally, Western blots were scanned and semi-quantitative analysis was performed using β-actin as a control for the protein loading. Each experiment was repeated at least three times [20].

### Small interfering RNA transfection

The hippocampal neurons with the Cx32 protein were transfected with Cx32 siRNA (siCx32) or the control siRNA (siMock) (Takara, Dalian, China) using Lipofectamine 2000 (Invitrogen, Carlsbad, CA), according to the method of Maxime [21] with slight modifications. The sequences corresponding to the Cx32 siRNA were: sense 5’-AAAACCGTCTTCACTGTCTTTCCTGTCTC-3’; antisense 5’-AAAAAGACAGTGAAGACGGTTCCTGTCTC-3’. Following transfection, cells were incubated at 37°C in a CO_2_ incubator for 48 h before being harvested for the assays described above.

### Construction of Cx32 adenoviral vectors

All recombinant adenovirus were constructed. Briefly, the full length of Cx32 cDNA was amplified and subcloned into pAdTrack-CMV, an adenoviral shuttle plasmid. The forward and reverse primers for Cx32 cDNA cloning were 5′-GAT AAG GTA CCC ATG AAC TGG ACA GGT TTG TAC ACC TTG-3′ and 5′-GAC TCG AGT CAG CAG GCC GAG CAG CGGTCG CTC TTT TCA G-3′, respectively [22]. We also constructed a recombinant adenovirus expressing green fluorescent protein (GFP), to assess infection efficiency. Then, the recombinant shuttle plasmids pAdTrack-CMV and pAdEasy-1 were homologously recombined in Escherichia coli strain BJ5183. After sequencing, recombinant adenoviruses were packaged and produced in 293A cells.

### Assessment of Connexin32 Activity

Uptake of a connexin hemichannel permeable dye (Lucifer yellow, LY) was measured to assess the extent of hemichannel opening in response to OGD alone and in the presence of TMP (10 µM and 20 µM) or siCx32. Neurons cultures were treated the same as described for the pharmacological analysis experiments. After media was removed for 15 seconds, it was replaced with 1 mL of test solution including 500 µM LY (Sigma Aldrich, St. Louis, MI). Sixty minutes after the addition of dye, cells were quickly rinsed twice with basal media containing 2 mM 1-octanol to remove residual LY while minimizing leakage of absorbed dye. LY fluorescence was read on a Victor3 1420 plate reader for 1 second per measure using 405 nm excitation and 535 nm emission filters. Three fluorescence measures were averaged for each data point [23].

### Statistical analysis

All data were expressed as mean±SD, and significance of differences was analyzed by one-way ANOVA followed by Dunnett’s Multiple Comparison test (SPSS 16.0 software). A value of *P*<0.05 were considered statistically significant.

## Results

### Neuron viability

**Figure 1:**
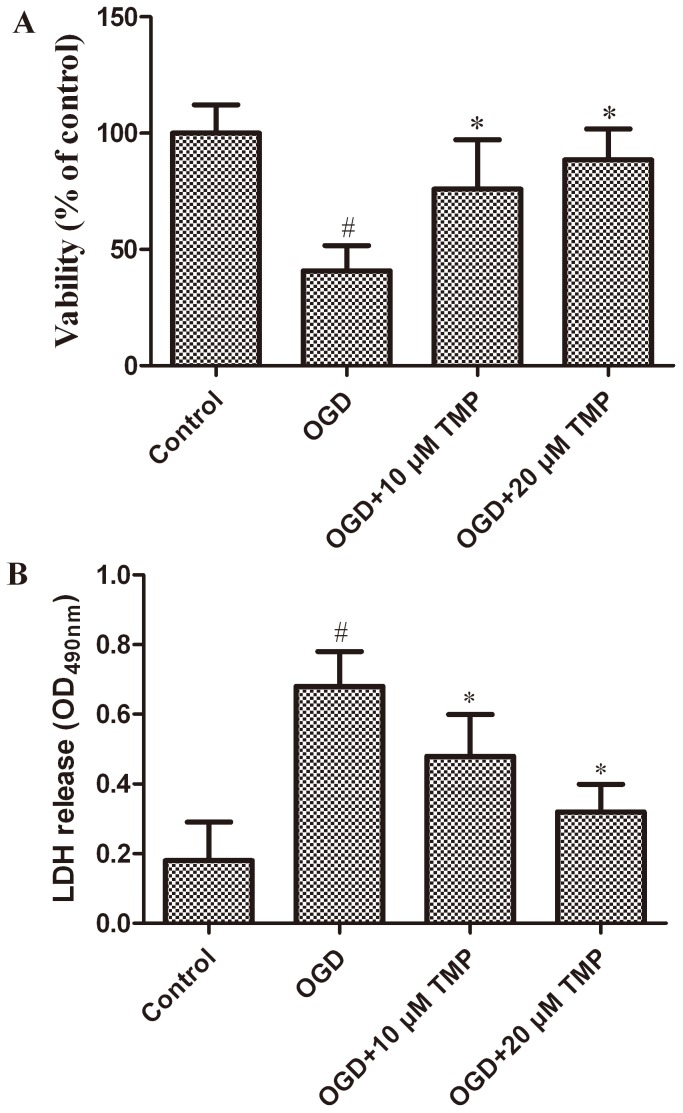
Effect of TMP on neurons viability. (A) MTT cell viability of normal control, OGD and TMP (10 and 20 µM) treatment groups (9 wells per group). Data are expressed as mean±SD; n = 9; # *P*<0.05 vs. control, *P<0.05 vs. OGD. (B) LDH release assay of normal control, OGD and TMP (10 and 20 µM) treatment groups. After OGD, increased LDH release was significantly attenuated by TMP. Data are expressed as mean±SD; n = 3, ^#^ *P*<0.05 vs. control, *P<0.05 vs. OGD.

The impact of TMP on hippocampal neuron viability was determined via MTT assay. The obtained results revealed that the viability of the neurons, to some extent, was promoted by TMP in a concentration-dependent manner (Fig. 1A). The neurons exposed to OGD indicated only 40.8% viability (% of Control group). TMP treatment at a concentration of 10 or 20 µM protected the neurons against cell death induced by OGD from 40.8% to 74.5% and 85.4%, respectively (*P*<0.05). On the other hand, the release of LDH into medium in hippocampal neurons increased after exposure to OGD, but pretreatment with TMP significantly decreased the LDH release in a concentration-dependent manner (Fig. 1B). These observations indicate that TMP could effectively, at least in part, reduced OGD-induced cell death.

### TMP treatment inhibits apoptosis in hippocampal neurons

**Figure 2:**
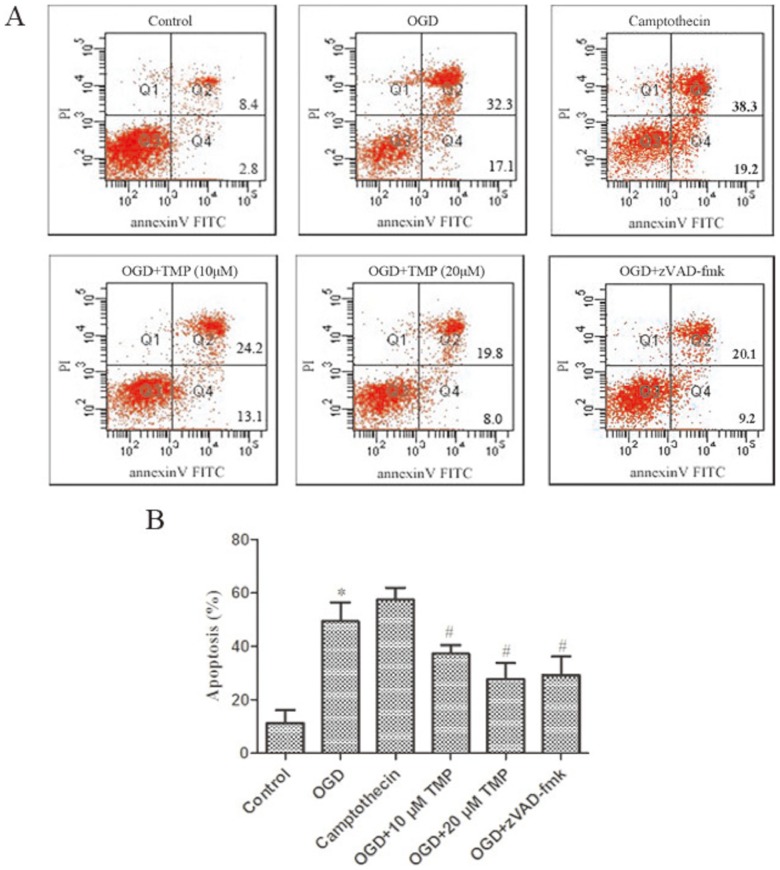
Effect of TMP on OGD-induced apoptosis in cultured hippocampal neurons. (A) Hippocampal neurons apoptosis was detected via PI staining and the Annexin V method. Data acquisition was conducted by collecting 20,000 cells per tube and the numbers of viable and dead cells were determined for each experimental condition. TMP (10 and 20 µM) and caspase inhibitor (zVAD-fmk 50 uM) were applied from OGD to the end of experiments. Controls and OGD group received the same amount of DMSO. The camptothecin group, neurons were exposure to 10 µM camptothecin used to experiment. (B) Columns show the mean of data obtained from three independent experiments. Bar mean SD. n = 3, * *P*<0.05 vs. control, ^#^ *P*<0.05 vs OGD.

Apoptosis of hippocampal neurons was detected by PI staining and the annexin V method and then analyzed by flow cytometry. TMP inhibited apoptosis and these effects were concentration dependent (Fig. 2). Under normal conditions, there was a very low level (11.2%) of neuronal apoptosis, but the percentage of apoptosis was significantly increased to 49.4% and 57.5% after OGD and camptothecin stimulation, and was reversed to 37.3%, 27.8% when treated with 10 µM, 20 µM TMP during OGD (*P*<0.05). The percentage of apoptotic cells did not differ significantly between the OGD group and camptothecin group. Caspase 3 activation was previously reported to be a reliable and sensitive marker of apoptosis [24]. We then examined the effect of caspase inhibitors on neurons apoptosis. A broad spectrum caspase inhibitor, zVAD-fmk at 50 uM, provided 20.1% reduction in the hippocampal neurons death compared the OGD group suggesting that apoptosis is the mode of death following OGD.

### TMP inhibited OGD-induced the expression of Cx32 in neurons

**Figure 3:**
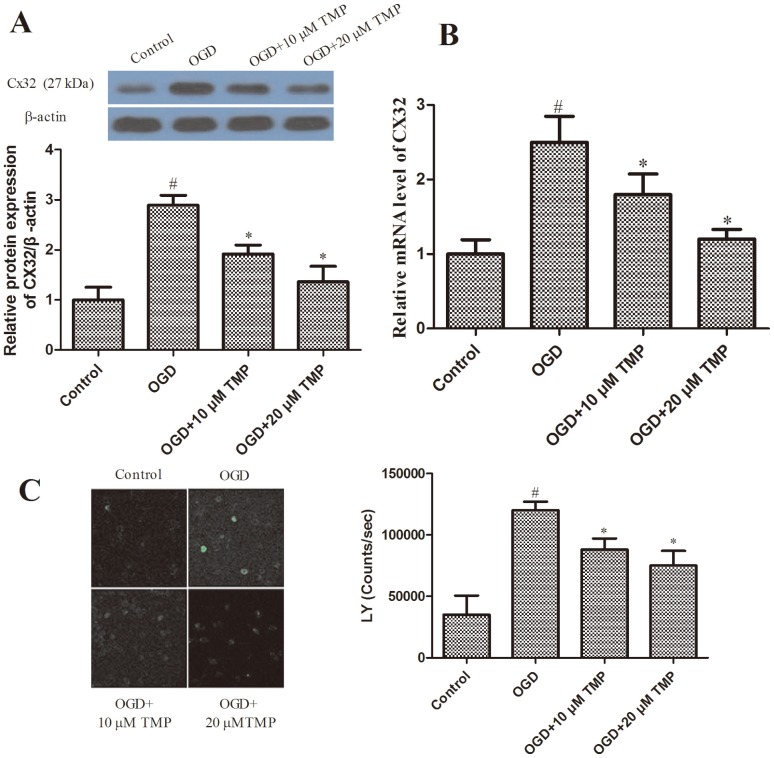
TMP treatment down-regulates the expression of Cx32 in hippocampal neurons. (A) The proteins Cx32 was determined using western blotting. β-actin was used as a loading control. (B) Cx32 mRNA expression is detected by qRT-PCR. All experiments were repeated at least three times. All data are means±SD. (n = 3) (#*P*<0.05 vs. control; *P<0.05 vs. OGD). (C) Uptake of the connexin permeable dye lucifer yellow (LY) was measured to confirm that Cx32 is activated during OGD and blocked by TMP. Values represent the counts of 9 wells per group±SD. (n = 3) (#*P*<0.05 vs. control; *P<0.05 vs. OGD).

The above results revealed that TMP could inhibit apoptosis in hippocampal neurons; however, the mechanism of TMP’s action is still unclear. A previous study showed global ischemia-induced increases in the gap junction protein Cx32 in hippocampus and enhanced vulnerability of Cx32 knock-out mice [9]. Therefore, we examined the role of Cx32 in TMP-inhibited apoptosis. As shown in Fig. 3A and B, as compared with the control group, OGD markedly up-regulated the expression of Cx32, however, TMP significantly inhibited OGD-induced the expression of Cx32 in neurons. To determine whether Cx32 is activated during OGD, uptake of the Cx permeable dye LY was measured following OGD. OGD significantly increased the uptake of LY (Fig. 3C), consistent with the up-regulation of CX32 expression in response to this stimulus. This increase in LY uptake was blocked by TMP during OGD.

### Effects of siRNA-Cx32 on TMP-stimulated neuron viability and apoptosis

**Figure 4:**
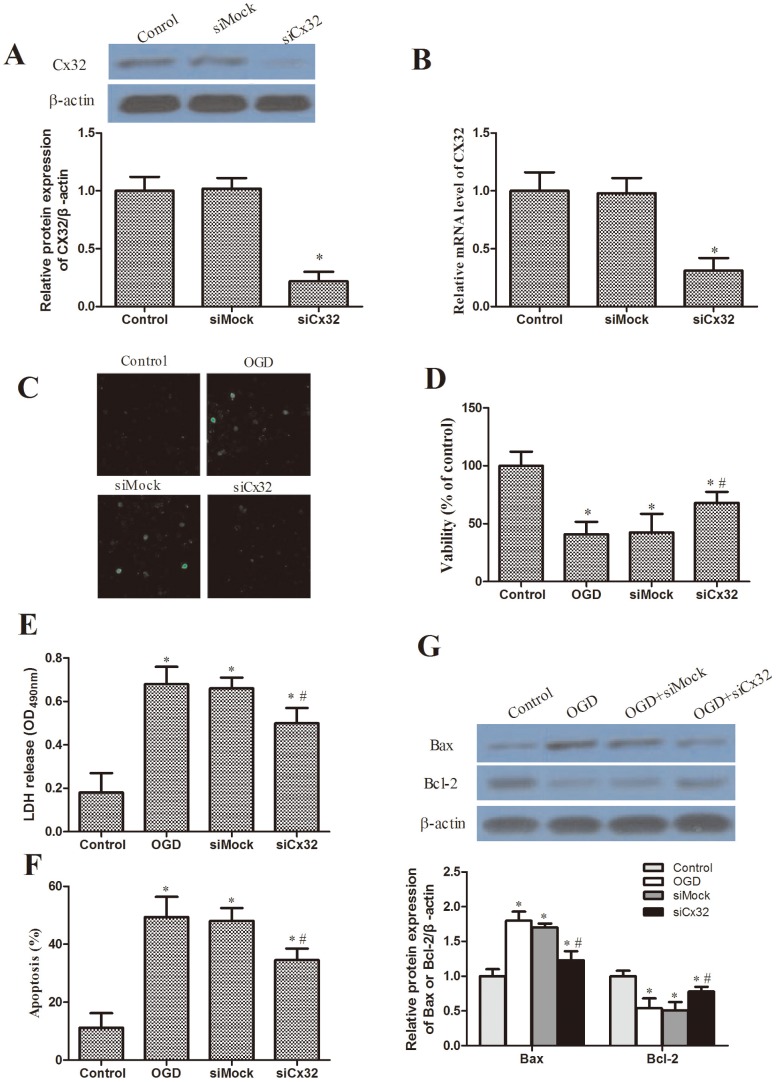
Inhibition of Cx32 expression and OGD -induced injury by siRNA-Cx32 in cultured hippocampal neurons. (A) Western blot analysis of Cx32 expression at 48 h after transfected with siCx32 and siMock. β-actin was used as a loading control. (B) Cx32 mRNA expression was detected by qRT-PCR at 48 h after transfected with siCx32 and siMock. All data are means±SD (*P<0.05 vs. control). (C) The photo images show that treatment of hippocampal neurons with siCx32 attenuated OGD-induced increase in LY-positive cells. (D) After OGD, MTT cell viability assay of normal control, siMock and siCx32 groups (9 wells per group). Data are expressed as mean±SD; *P<0.05 vs. control, #*P*<0.05 vs. OGD. (E) LDH release assay of normal control, OGD and TMP (10 and 20 µM) treatment groups. Data are expressed as mean±SD; *P<0.05 vs. control, #*P*<0.05 vs. OGD. (F) Hippocampal neurons apoptosis was detected via PI staining and the Annexin V method. Data acquisition was conducted by collecting 20,000 cells per tube and the numbers of viable and dead cells were determined for each experimental condition. Columns show the mean of data obtained from three independent experiments. Bar mean SD. * *P*<0.05 vs. control, ^#^ *P*<0.05 vs OGD. (G) The proteins Bcl-2 and Bax were determined using western blotting with corresponding antibodies. β-actin was used as an internal control. All experiments were repeated at least three times.

Since Cx32 expression was down-regulated in hippocampal neurons treated with TMP, we assumed that Cx32 might play a significant role in the activity of TMP. So we next examined whether Cx32 affected the impact of TMP on viability and apoptosis in stimulated neurons. To measure Cx32 functions in vitro, a siRNA experiment was performed in neurons. As shown in Fig 4A and 4B, transfection of siCx32 into hippocampal neurons significantly reduced the Cx32 expression, but a non-silencing control siRNA had no such effect. Cx32 siRNA inhibited OGD-increased the uptake of LY (Fig. 4C). Moreover, Cx32 siRNA significantly attenuated the OGD-induced reduction in cell viability and increase in LDH release (Fig. 4D and E). In addition, the neuron apoptosis rate was decrease in the siCx32-transfected neurons and neuron apoptosis was suppressed by activating Bcl-2 expression and inhibiting Bax expression (Fig 4F and G).

### Effects of Cx32 overexpression on neuron viability

**Figure 5:**
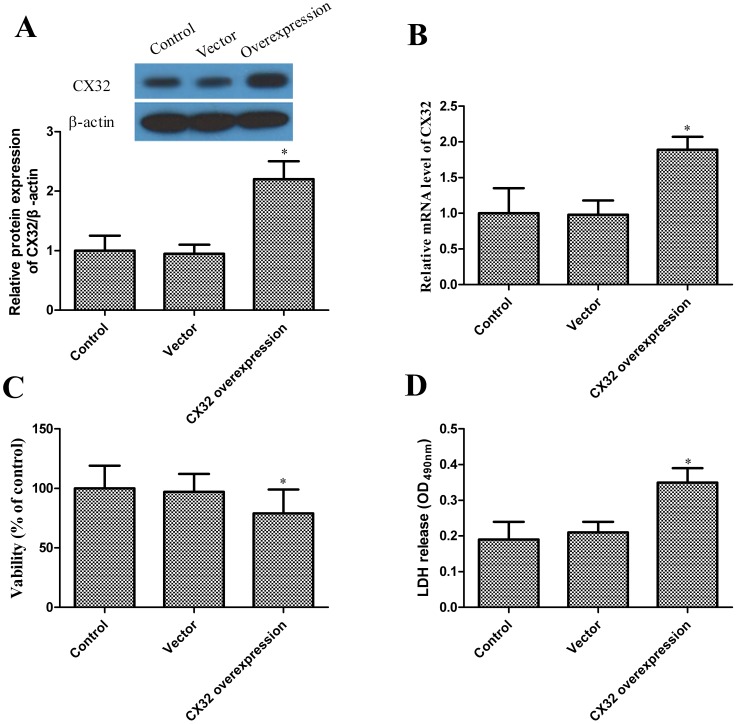
Effects of Cx32 overexpression on neuron viability. (A) Western blot analysis of the protein levels of Cx32 in neuron, β-actin was used as a loading control. (B) Cx32 mRNA expression levels expression is detected by RT-PCR. (C) After OGD, MTT cell viability assay of normal control, Vector and Cx32 overexpression groups. (D) LDH release of normal control, Vector and Cx32 overexpression groups. All experiments were repeated at least three times. Data are expressed as mean±SD; *P<0.05 vs. control.

In order to further confirm the role of Cx32, a Cx32 overexpression experiment was performed in hippocampal neurons. Fig. 5A and B show that Cx32 expression significantly increased following transfection of adenoviral vector with Cx32 into hippocampal neurons. In addition, there was a decrease in neurons viability and increase in LDH release (Fig. 5C and D), suggesting that increased Cx32 expression is related to neurons viability.

### Effects of TMP on MAPKs pathway

**Figure 6:**
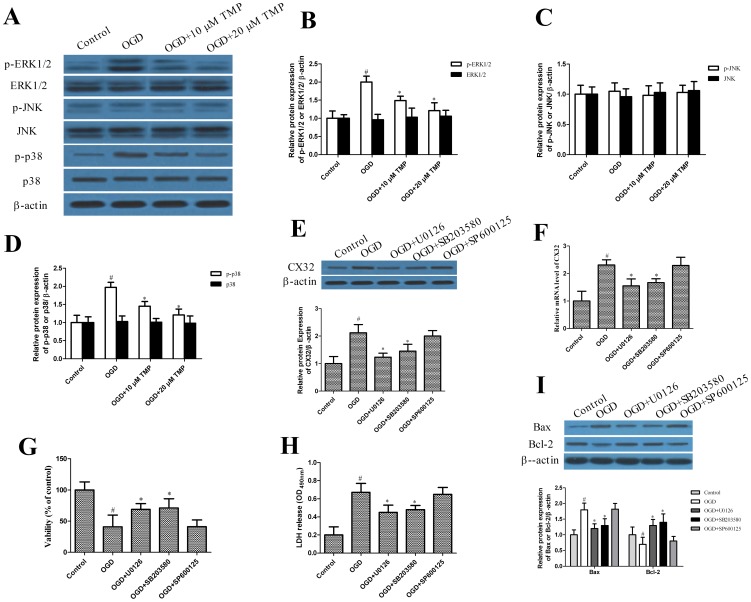
Effects of TMP on MAPKs pathway. (A) The proteins ERK, p-ERK, p38, p-p38, JNK and p-JNK were determined using western blotting with corresponding antibodies. β-actin was used as an internal control. (B, C, D) Data are expressed as mean±SD; #*P*<0.05 compared with control; *P<0.05 compared with OGD. (E) OGD-induced up-regulation of Cx32 expression was inhibited by both the ERK inhibitor U0126 (5 µM) and the p38 inhibitor SB203580 (5 µM), but not by the JNK inhibitor SP600125 (5 µM). The Cx32 protein was determined using western blotting. β-actin was used as a loading control. (F) Cx32 mRNA expression was detected by qRT-PCR. All data are means±SD (#*P*<0.05 vs. control; *P<0.05 vs. OGD/R). (G) MTT cell viability of normal control, OGD and U0126 (5 µM), SB203580 (5 µM) and SP600125 (5 µM) treatment groups (9 wells per group). Data are expressed as mean±SD; #*P*<0.05 vs. control, *P<0.05 vs. OGD. (H) LDH release assay normal control, OGD and U0126 (5 µM), SB203580 (5 µM) and SP600125 (5 µM) treatment groups. Data are expressed as mean±SD; #*P*<0.05 vs. control, *P<0.05 vs. OGD (I) The proteins Bcl-2 and Bax were determined using western blotting with corresponding antibodies. β-actin was used as an internal control. All experiments were repeated at least three times.

Mitogen-activated protein kinases (MAPKs) regulate diverse cellular programs [25] and there is some evidence showed that ERK1/2 and p38 kinase signaling pathway modulate gap junction intercellular communication in several kinds of cell lines [26]. To clarify the roles of the MAPK signaling pathway in TMP-upregulated the expression of Cx32, we determined the expression of ERK1/2, p-ERK1/2, JNK, p-JNK p38 and p-p38 by western blot and the effects of their inhibitors. Fig. 6A, B, C and D show that OGD increased the levels of phosphorylated ERK1/2 and p38, which were inhibited by treatment of TMP. while phosphorylated JNK did not significantly change. The level of ERK1/2, p38 and JNK protein was statistically similar in all groups. Furthermore, the ERK1/2 inhibitor U0126 and the p38 inhibitor SB203580 significantly inhibited Cx32 up-regulation after OGD, but the JNK inhibitor SP600125 did not (Fig.6E and F). U0126 and SB203580, but not SP600125, also attenuated OGD -induced hippocampal neurons injury. (Fig.6G, H and I).

## Discussion

Ischemic brain injury is a leading cause of dementia, disability and death worldwide. After the onset of brain ischemia, a series of events lead ultimately to the death of neurons [27], [28]. There have been a number of attempts to develop neuroprotectants for brain ischemia, such as FGF-2, a brain-derived neurotrophic factor that has been tested for its ability to rescue neurons from ischemic cell death [29], [30]. However, therapies for ischemic brain injury are far from satisfactory and many of these attempts have failed. Therefore, there remains an urgent need to find substances or drugs that can limit or reverse ischemic injury.

It has been reported that TMP exhibited neuroprotective and anti-inflammatory effects in rats subjected to permanent cerebral ischemia [31]. The present study demonstrated that TMP protects neurons against cell apoptosis processes induced by OGD. These results seem to be in agreement with literature data showing TMP possesses good neuroprotective effects against ischemic brain injury. Intravenous administration of TMP before ischemia-reperfusion injury may have protective effects on intestinal tissue [32]. However, the exact mechanism of TMP in neuroprotection is poorly understood.

Cxs are the family of membrane proteins that constitute gap junctions (GJs), channel-like structures that connect the cytoplasm of neighboring cells and facilitate intercellular communication [33], [34]. Cx32 was a member of the Cx protein family which was found to act as key players in charge of hepatocyte proliferation and cell death [35], [36]. On the one hand, Mathieu *et al* previously investigated Cx32 hemichannels facilitate the apoptotic-to-necrotic transition, which typically occurs in the final stage of hepatocellular apoptosis [37]. On the other hand, Isao *et al* demonstrated that Cx32 protects against acetaminophen-induced hepatic centrilobular necrosis in mice [38]. Although seemingly paradoxical, these results may all be correct depending on the cells examined and the type of stimulation. It is now well established that there is a closeness relationship between Cx32 and ischemic brain injury. Moreover, Cx32 expression is significantly increased in ischemic lesions in aged human brain [39]. However, whether TMP protected ischemic injury in hippocampal neurons is associated with Cx32 remains unclear.

It has been reported that OGD enhances gap junction coupling and promotes bystander cell killing *in vitro* trauma model [40]. Introducing gap junction proteins (Cxs) into cell lines has been shown to enhance bystander cell death [41]. In addition, gap junction blocker carbenoxolone could reduce OGD-induced neuronal death in hippocampal slice cultures [42]. Consistent with these reports, in this study, we found the quantitative inverse relationship between Cx32 expression and hippocampal neuron viability. OGD insult caused a remarkable increase in the mRNA and protein levels of Cx32 and TMP significantly inhibited the Cx32 expression. To determine whether Cx32 function is relevant to neuron biological activities, we performed Cx32 siRNA experiments and measured neuron viability and apoptosis. Knockdown of Cx32 expression by siRNA attenuated OGD-induced cell injury, which suggest that TMP suppresses OGD-induced Cx32 expression and cell apoptosis. In contrast, Oguro et al reported that global ischemia selectively increases Cx32 protein expression in parvalbumin-positive inhibitory interneurons in CA1, and Cx32-null mice exhibit enhanced vulnerability to global ischemia-induced damage, which suggest Cx32 may play a critical role in protection and survival of CA1 interneurons after global ischemia [9]. However, chronic deficiency of Cx32 in the knock out mice may induce compensatory mechanisms that could account for this discrepancy. Further experiments are needed to clarify this issue.

Another important finding in the present study is clarification of the signaling pathway. Yang *et al* reported that ERK1/2 and p38 MAP kinase signaling pathway may be closely related functionally to regulate gap junction in rat neuronal stem cell-derived cells [43]. Consistent with their finding, we found that increased phosphorylation of ERK1/2 and p38, but not JNK, mediated the up-regulation of Cx32 induced by OGD. TMP attenuated ERK1/2 and p38 phosphorylation after OGD, and the inhibitors of ERK1/2 and p38 attenuated Cx32 up-regulation and hippocampal neurons injury after OGD.

In summary, the present work shows that TMP suppresses OGD induced connexin32 expression and cell apoptosis throughout the ERK1/2 and p38 MAPK signaling pathways. The present findings suggest that TMP demonstrated a significant effect on ischemic brain injury via protecting hippocampal neurons.
